# *Embrace Pain*—A Randomized Controlled Trial of Online Acceptance and Commitment Therapy for Painful Chemotherapy‐Induced Peripheral Neuropathy in Cancer Survivors

**DOI:** 10.1002/pon.70608

**Published:** 2026-09-19

**Authors:** Daniëlle L. van de Graaf, Paul Lodder, Tom Smeets, Marije L. van der Lee, Hester R. Trompetter, Karlein M. G. Schreurs, Elin Børøsund, Lise Solberg Nes, Floortje Mols

**Affiliations:** ^1^ Department of Medical and Clinical Psychology CoRPS—Center of Research on Psychological Disorders and Somatic Diseases Tilburg University Tilburg the Netherlands; ^2^ Tranzo Tilburg School of Social and Behavioral Sciences Tilburg University Tilburg the Netherlands; ^3^ Department of Methodology and Statistics Tilburg University Tilburg the Netherlands; ^4^ Centre for Psycho‐Oncology Scientific Research Department Helen Dowling Institute Bilthoven the Netherlands; ^5^ Department of Psychology, Health & Technology Centre for eHealth & Well‐being Research University of Twente Enschede the Netherlands; ^6^ Department of Digital Health Research Division of Medicine Oslo University Hospital Oslo Norway; ^7^ Department of Nursing and Health Sciences Faculty of Health and Social Sciences University of South‐Eastern Norway Drammen Norway; ^8^ Department of Psychiatry and Psychology College of Medicine and Science Mayo Clinic Rochester Minnesota USA; ^9^ Faculty of Medicine Institute of Clinical Medicine University of Oslo Oslo Norway; ^10^ Department of Research Netherlands Comprehensive Cancer Organisation (IKNL) Utrecht the Netherlands

**Keywords:** acceptance and commitment therapy (ACT), cancer survivorship, chemotherapy‐induced peripheral neuropathy (CIPN), online self‐help intervention, pain interference

## Abstract

**Introduction:**

Chemotherapy‐induced peripheral neuropathy (CIPN) is a common and persistent side effect of chemotherapy, with up to 30% of patients reporting symptoms 6 months post‐treatment. Painful CIPN can impair sleep, mood, and physical functioning. We evaluated “Embrace Pain,” an online Acceptance and Commitment Therapy (ACT) based self‐help intervention that may be of support for cancer survivors with chronic painful CIPN.

**Methods:**

The Embrace Pain Randomized Controlled Trial (RCT) compared ACT with a waiting list condition (WLC) using a two‐armed randomized design. Adults (≥ 18 years) with painful CIPN ≥ 6 months post‐chemotherapy were randomized to an 8‐week guided online ACT program or WLC. Primary outcome was pain interference (Multidimensional Pain Inventory, MPI). Secondary outcomes included pain catastrophizing, psychological flexibility, CIPN severity, pain intensity, and quality of life (QoL). Linear mixed models and criteria for clinically significant change were used to evaluate outcomes from baseline to 3‐month follow‐up.

**Results:**

112 participants were included (57 ACT, 55 WLC). No significant treatment effect was observed for the primary outcome, pain interference, with ACT not outperforming the WLC over time. However, more ACT participants showed clinically significant improvement in pain interference at 3‐month follow‐up (47.4% vs. 23.6%) than WLC participants, and exploratory analyses showed greater reductions in pain catastrophizing in the ACT group. No between‐group differences were found for psychological flexibility, CIPN severity, pain intensity, or QoL.

**Discussion:**

Online ACT did not demonstrate a significant treatment effect on pain interference in those with painful CIPN. Exploratory findings suggest potential benefits for pain catastrophizing and clinically meaningful improvement in pain interference for some participants. Larger studies are needed to confirm these findings.

## 1 Introduction

A common side effect of chemotherapy is chemotherapy‐induced peripheral neuropathy (CIPN) [1], which involves damage to the peripheral nerves caused by various chemotherapeutic agents [2]. Symptoms of CIPN may include tingling, numbness, pain, or cramps in hands, arms, feet and legs [3, 4, 5]. Approximately 30% of people continue to experience these symptoms 6 months after completing chemotherapy [1, 6, 7, 8, 9, 10]. Even up to 11 years after completing chemotherapy, patients with CIPN report diminished quality of life (QoL) due to persistent symptoms and associated limitations [6, 11, 12, 13, 14]. Some patients also experience persistent pain resulting from their CIPN. Painful CIPN is also often linked to greater pain interference, defined as the patient's perceived impact of pain on daily life [15]. In addition, painful CIPN has been associated with impaired sleep, anxiety, depression, fatigue, symptom distress, and reduced physical functioning [16]. The American Society of Clinical Oncology (ASCO) guidelines indicate that duloxetine may provide modest relief for CIPN–related pain, but further effective treatment options remain limited.

Recently, an online self‐management intervention based on Acceptance and Commitment Therapy (ACT), called “Embrace Pain” for patients with painful CIPN was developed [17]. ACT is a cognitive behavioral treatment (CBT) that promotes acceptance of pain as an alternative to pain avoidance, helping patients shift their focus toward engaging in personally meaningful life activities [18]. An earlier study in patients with CIPN demonstrated positive outcomes of an online CBT self‐management intervention, including reductions in pain severity and interference, and improvements in neuropathy‐related symptoms [19]. ACT is deemed an evidence‐based treatment for chronic pain by the American Psychological Association [20], and many Dutch rehabilitation centers adopted ACT as one of the primary modes of treatment for chronic pain [21]. The Embrace Pain intervention is expected to yield favorable results, given the demonstrated benefits of ACT for chronic pain, both in traditional face‐to‐face settings [22, 23, 24, 25] as well as online [26]. Recent evidence further supports this, as an overview of systematic reviews with meta‐analysis of randomized controlled trials (RCTs) showed that ACT can lead to small to moderate improvements in pain interference, psychological flexibility, and QoL in patients with chronic pain [27].

Delivering psychological interventions online offers important advantages, including lower costs by reducing the need for face‐to‐face therapist time, greater accessibility and convenience for patients who can participate from home, and reduced stigma by allowing individuals to engage in psychological support privately and at their own pace [19, 28, 29]. Given the existing evidence supporting both ACT and CBT‐based online interventions for chronic pain and CIPN, it is plausible that online ACT may help patients with chronic painful CIPN reduce pain interference, even when pain symptoms persist. However, despite growing evidence for psychological interventions in chronic pain, relatively few studies have specifically focused on neuropathic pain populations, including patients with painful CIPN. Consequently, evidence regarding the effectiveness of psychological interventions in these populations remains limited.

Against this background, the effectiveness of online ACT self‐management interventions for chronic painful CIPN remains largely unknown. Furthermore, intervention effects may vary according to baseline patient characteristics [30] and intervention characteristics, such as the availability of guidance [31, 32]. Therefore, the present study had three objectives: (1) To evaluate the effectiveness of an online ACT self‐help intervention for reducing pain interference in cancer survivors with chronic painful CIPN in a randomized controlled trial, compared with a waiting list condition (WLC), and to assess its effects on relevant secondary outcomes including pain intensity, psychological distress, quality of life, and pain‐related functioning. (2) To examine which baseline demographic, clinical, and psychosocial factors may moderate the effectiveness of the online ACT‐based self‐help intervention. (3) To examine the extent to which effects differ between guided and unguided versions of the online ACT self‐help intervention, and to determine whether intervention adherence influences or moderates treatment outcomes.

## 2 Methods

### 2.1 Study Design

A two‐armed parallel, non‐blinded RCT was conducted to evaluate the effectiveness of an online ACT‐based self‐help intervention compared with WLC in reducing pain interference among cancer survivors with chronic painful CIPN. Study participation lasted approximately 8 months, during which six questionnaires were administered: at inclusion and baseline (T0), early intervention (3 weeks; T1), mid‐intervention (6 weeks; T2), post‐treatment (T3; end of the 8‐week intervention), and at 3‐month (T4) and 6‐month (T5) post‐treatment follow‐up. The study was approved by a certified Medical Research Ethics Committee (Medisch Ethische Toetsingscommissie Brabant; reference number NL78436.028.21/P2133), prospectively registered in the ClinicalTrials.gov registry under identifier NCT05371158, and a detailed study protocol has been published [33].

### 2.2 Study Population

Eligibility criteria were: (a) age ≥ 18 years, (b) clinician‐diagnosed or self‐identified bilateral painful sensations including aching, burning, “pins‐and‐needles,” shock‐like pains, painful tingling, numbness, or cramps in the feet/legs and/or hands/arms for ≥ 3 months (self‐reported), (c) pain intensity ≥ 3 on an 11‐point Numeric Rating Scale (NRS‐11), (d) pain not present prior to chemotherapy (self‐reported), (e) chemotherapy completed ≥ 6 months before study entry (self‐reported), and (f) a score ≥ 1 on the EORTC QLQ–CIPN20 scale [34].

Because no universally accepted diagnostic gold standard for painful CIPN exists in survivorship research, eligibility was based on a combination of prior chemotherapy exposure, characteristic neuropathic symptoms, pain intensity, symptom duration, and EORTC QLQ‐CIPN20 scores.

Exclusion criteria were: (a) current psychological treatment related to cancer, pain, or psychological disorders, (b) planned chemotherapy during the study period, (c) no internet access, (d) insufficient time to follow the intervention (≥ 2 h per week), and (e) reading or learning difficulties in Dutch.

### 2.3 Recruitment

Patients were recruited from December 2021 till October 2023. Five hospitals supported recruitment, three of which directly approached eligible patients by recruitment letters. Flyers were distributed to outpatient clinics across the Netherlands and cancer support centers in both the Netherlands and Belgium. Additional recruitment occurred online via patient platforms, patient organizations, and professional associations. Study presentations were also delivered to patient groups and relevant professional meetings. Interested individuals could register via the online study portal. After providing signed informed consent, patients completed an eligibility questionnaire. If desired, their general practitioner or medical specialist was informed of participation.

### 2.4 Randomization

Patients were randomly assigned to the ACT or WLC group. Stratification by age and sex was applied using block‐wise randomization generated by an online randomization tool, with a fixed block size of 26 participants. Within each block, 13 participants were randomly assigned to each condition (coded as 1 or 2), ensuring equal allocation per block. For each subsequent block of 26 participants, this procedure was repeated.

### 2.5 Intervention

#### 2.5.1 Experimental Condition (ACT)

The experimental condition consisted of a guided online ACT intervention, developed using a patient‐centered approach [17, 33], delivered through a secure eHealth platform that could be accessed via a webpage or mobile app, including six modules accessible over 8 weeks. All communication and data storage complied with GDPR regulations, and the platform was ISO 27001 and NEN 7510 certified to ensure confidentiality and data security. The program consisted of psychoeducation and all six core ACT processes: acceptance, cognitive defusion, mindfulness, self‐as‐context, values clarification, and committed action, with ACT seeking to promote acceptance of pain as an alternative to pain avoidance, helping patients to shift their focus toward the performance of personally valuable activities [18]. Participants had access to various therapeutic formats through the study portal, such as text, metaphors, experiential exercises, illustrations, and audio files, designed to reduce pain avoidance and promote pain acceptance. The intervention could be completed at any time and place, offering full flexibility.

Participants were encouraged to complete approximately one module per week over the 8‐week intervention period. As specified in the exclusion criteria, participants had to be able to dedicate at least 2 hours per week to the intervention, resulting in an intended minimum intervention exposure of approximately 16 hours. Participants also received therapeutic email guidance from master's students in Psychology and one of the researchers (DG), supervised by a licensed healthcare psychologist. Guidance was intentionally low‐intensity and optional. Participants could contact a coach by email throughout the intervention and received feedback on questions or reflections they submitted, but no scheduled therapist sessions were provided. A detailed description of the “Embrace Pain” intervention can be found in the protocol [33] and the development [17] papers.

#### 2.5.2 Waiting List Condition (WLC)

The intervention group received the online ACT program over a period of 8 weeks. A follow‐up assessment (T4) was conducted 3 months after completion of the intervention, corresponding to 5 months after baseline. Participants in the waiting list control group (WLC) did not receive any intervention during this 5‐month period. After the intervention group had completed the T4 assessment, WLC participants were offered access to the unguided version of the online ACT intervention. The final assessment (T5) for all participants took place 6 months after intervention.

### 2.6 Outcomes

#### 2.6.1 Primary Outcome

Pain interference was measured by means of the Interference subscale of the Multidimensional Pain Inventory, Dutch language version (MPI‐DLV) [15, 35]. The subscale consists of 11 items rated on a 7‐point Likert scale ranging from 0 (no change) to 6 (a lot of change). It assesses psychosocial aspects of chronic pain (e.g., functioning in work, recreational and social activities). The Dutch version has demonstrated good psychometric properties [35]. In the current study, internal consistency was high, with Cronbach's alpha values ranging from 0.89 to 0.92 across the 5 measurement occasions.

#### 2.6.2 Secondary Outcomes

Quality of life was measured using the European Organization for Research and Treatment of Cancer Quality of Life Questionnaire Core‐30 (EORTC QLQ‐C30), which includes a global health status/overall quality of life scale, five functional subscales (physical, role, emotional, cognitive, and social functioning), and several symptom scales [36]. CIPN symptom severity was assessed using the EORTC QLQ–CIPN20 that comprises three subscales (i.e., sensory, motor, and autonomic symptoms) and a total score [37, 38]. Pain intensity was evaluated using the NRS‐11 [39]. Psychological distress was measured using the Hospital Anxiety and Depression Scale (HADS), consisting of anxiety and depressive symptom subscales [40, 41]. Pain catastrophizing was measured using the Pain Catastrophizing Scale (PCS), which includes rumination, magnification, and helplessness subscales [42]. Psychological flexibility was assessed through the Psychological Inflexibility in Pain Scale (PIPS), containing avoidance and cognitive fusion subscales [43, 44]. Mindfulness was evaluated with the Freiburg Mindfulness Inventory (FMI), including presence and acceptance subscales [45]. Values‐based living was measured using the Engaged Living Scale (ELS), comprising value‐based living and life fulfillment subscales [46]. Information on the validity and reliability of these measures is provided in the study protocol [33]. Supporting Information S1: Table S1 reports the estimated reliability coefficients for each measurement occasion. All scales showed acceptable reliability (estimates ≥ 0.7) except for the autonomic symptom subscales of the CIPN20 and the QLQ‐C30 cognitive functioning subscale at later timepoints.

#### 2.6.3 Other Measurements

Socio‐demographic characteristics, clinical cancer and CIPN‐related information, psychological problems (i.e., depressive symptoms and anxiety), and comorbidities were assessed.

### 2.7 Sample Size

Based on previous studies evaluating online and face‐to‐face ACT interventions for chronic pain [47, 48], a between‐group effect size of *d* = 0.50 was assumed. A total of 51 participants per group with complete follow‐up data were required to achieve 80% power at a two‐sided significance level of 0.05. Anticipating a dropout rate of 30% [33], the target sample size was set at 146 participants.

A total of 112 participants were ultimately randomized, resulting in lower statistical power than originally planned. All randomized participants were included in the intention‐to‐treat analyses. Missing data were addressed using likelihood‐based estimation in the linear mixed models and imputation procedures for the clinical significance analyses, but these approaches cannot fully compensate for reduced sample size and attrition during follow‐up.

#### 2.7.1 Statistical Analysis

Baseline characteristics were expressed as mean (SD) for continuous variables and *N* (%) for categorical variables. Significant differences in baseline characteristics between the ACT and WLC conditions were checked by performing one‐way ANOVAs and chi‐square tests. These models were also used to test for differences in baseline characteristics between completers and dropouts, while applying a Holm‐Bonferroni correction for multiple testing. All analyses were conducted using SPSS statistics (version 29). *p*‐values smaller than 0.05 were considered statistically significant.

#### 2.7.2 Primary Analyses

Effects on all primary and secondary outcomes were evaluated using intention‐to‐treat analyses with linear mixed models. Given that participants in the WLC condition gained access to the intervention after the 3‐month follow‐up, the primary ACT versus WLC effectiveness analyses did not include the 6‐month follow‐up. Accordingly, the model assessed changes from baseline (T0) to post‐intervention (T3) and the 3‐month‐follow‐up (T4). The model included fixed effects for treatment (2 levels), time (3 levels), and their first‐order interaction. The dependency in the repeated measurements within individual was modeled using an unstructured residual covariance structure. Parameters were estimated using restricted maximum likelihood estimation (REML), allowing inclusion of all available data from participants who dropped out during follow‐up. This approach provides unbiased estimates under the assumption that data are missing at random. Because the validity of this assumption cannot be directly tested, the potential impact of non‐random attrition was considered when interpreting the findings. At post‐intervention and follow‐up, effect sizes (Cohen's *d*) were calculated using estimated marginal means and standard deviations. Effect sizes of 0.80, 0.50, and 0.20 were considered as large, moderate and small, respectively [49].

#### 2.7.3 Clinical Significance

Clinically significant change was evaluated in accordance with The Initiative on Methods, Measurement, and Pain Assessment in Clinical Trials (IMMPACT) recommendations [50]. Missing values were imputed using the Expectation Maximization algorithm. For each condition, the proportions of participants showing clinically significant improvement were calculated, and chi‐square tests were used to assess between‐group differences. These calculations were performed for (a) pain interference (MPI—Interference), (b) pain intensity (NRS), and (c) CIPN symptom severity (EORTC QLQ—CIPN 20). A reduction of ≥ 0.6 standard deviation on the MPI‐Interference scale was considered clinically meaningful, following IMMPACT guidelines [50]. For pain intensity (NRS), a decrease of ≥ 20% was considered indicative of minimal to moderate improvement. As no specific IMMPACT guidelines exist for the CIPN20, a distribution‐based threshold was used: a decrease of ≥ 1 standard error of measurement (based on baseline CIPN scores) was considered clinically significant [50]. Because the study protocol did not specify the timepoint at which clinical significance should be evaluated, we conducted the analysis both at post‐treatment and 3‐month follow‐up, while adjusting the two resulting *p*‐values using a Holm‐Bonferroni correction for multiple testing.

### 2.8 Secondary Analyses

Because drop‐out and non‐adherence are relatively common in online interventions [51], secondary per‐protocol analyses were conducted to examine effects among participants who adhered to the intervention, defined as completing all sessions and self‐reporting ≥ 2 hours per week of engagement.

To identify predictors of treatment effects (objective 2), the steps from a previous study on predictors of change during CBT for chronic pain were followed [52]. Moderation analyses were applied using ANCOVA, with pain interference at the 3‐month follow‐up (T4) as the dependent variable. Baseline pain interference was included as covariate. Predictor variables consisted of baseline patient characteristics and their interaction with treatment conditions. Predictor variables included sex, age, educational level, number of comorbidities, time since CIPN onset, CIPN symptom severity, quality of life (EORTC QLQ‐C30 domains), pain intensity (NRS), psychological distress (HADS), psychological inflexibility (PIPS), mindfulness (FMI‐NL), value‐based living (ELS), and pain catastrophizing (PCS). These variables were selected based on theoretical and empirical considerations.

To assess differences in treatment effects between the guided and unguided versions of the intervention (objective 3), we applied the same linear mixed‐model approach described above. For participants in the ACT group, change from baseline to 3‐month follow‐up was used as the dependent variable. For participants who crossed over from the WLC to the unguided self‐help intervention, change from the 3‐month to the 6‐month follow‐up was used as the corresponding dependent measure.

## 3 Results

**FIGURE 1:**
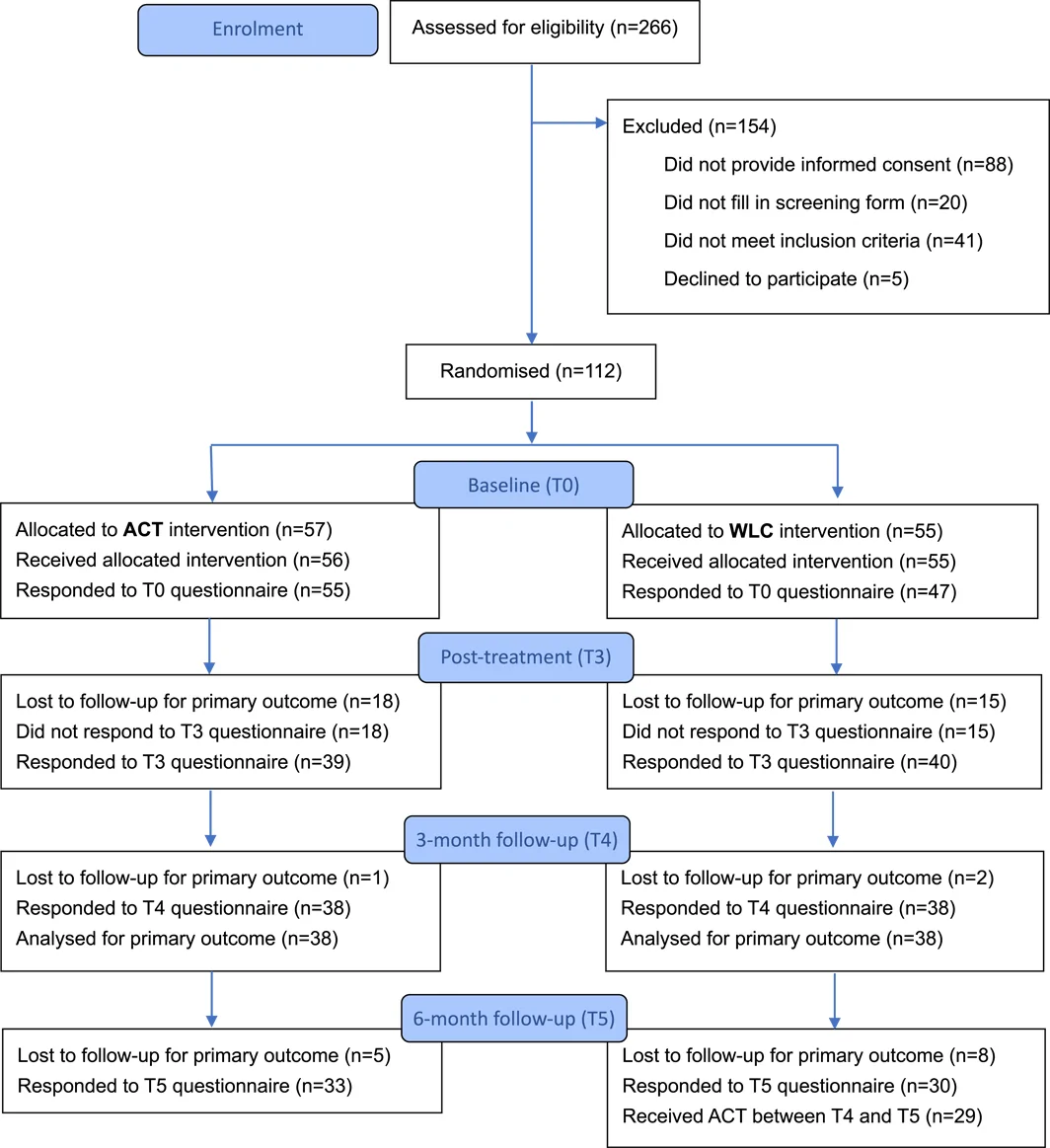
CONSORT flow diagram of the RCT comparing ACT‐based online self‐help with a waitlist control.

After a period of 22 months, a total of 112 patients were included, of which 57 were randomly assigned to the ACT condition and 55 to the WLC condition. Although the planned sample size was not achieved, the primary analyses were conducted as prespecified. As shown in the flow chart (Figure 1), dropout over the follow‐up period was considerable. Regarding the primary outcome measure (MPI), data at post‐test (T3) were available for 79 (39 ACT and 40 WLC) of the 112 participants (71%). This decreased to 76 participants (38 ACT and 38 WLC; 68%) at the 3‐month follow‐up (T4) and 64 participants (33 ACT and 29 WLC; 57%) at the 6‐month follow‐up (T5). Based on the 76 participants available at T4, rather than the 102 participants with complete follow‐up (derived from the original recruitment target of 146 participants assuming 30% attrition, a post hoc sensitivity power analysis indicated that the study still had 80% power to detect between‐group differences of approximately *d* = 0.65. However, the reduced sample size may have limited our ability to detect smaller treatment effects and substantially reduced power for the per‐protocol analyses. Of the 39 participants who completed the T3 questionnaire in the ACT condition, 36 participants (92%) made use of at least one contact with the online coach, whereas two participants (5%) did not make use of the available guidance and one participant (2.5%) did not respond to this question.

Supporting Information S1: Table S2 presents the results of several ANOVAs and chi‐square tests to estimate differences in baseline characteristics between participants who completed all assessments (T5 completers) versus those who discontinued participation during follow‐up (dropouts). Findings indicate statistically significant differences in several characteristics. On average, dropouts were slightly younger, more often female, had lower ELS life fulfillment scores, higher HADS depression scores, and lower scores on the EORTC QLQ‐C30 global quality of life, role, cognitive and social functioning, and higher fatigue. Nevertheless, none of these differences remained significant after applying the Holm‐Bonferroni correction for multiple testing. Little's MCAR test did not yield statistically significant results, suggesting that data were missing completely at random.

Table 1 shows the baseline characteristics for patients randomized to the ACT and WLC groups. On average, participants in the ACT group showed higher mean scores on ELS and on the PCS helplessness subscale. Nevertheless, given the randomization procedure and the increased likelihood of chance findings when examining multiple baseline variables, these differences are assumed to reflect random variation rather than systematic group imbalance.

**TABLE 1:** Baseline characteristics of the 57 participants randomized into the online self‐help ACT group and the 55 participants in the waiting list control (WLC) group.

Patient characteristic	Online self‐help ACT (*n* = 57)	Waiting list control WLC (*n* = 55)
Female sex	37 (65%)	41 (75%)
Age	63.6 (11.6)	63.8 (9.9)
Highest completed education		
Primary/secondary school	6 (11.1%)	7 (14.6%)
Vocational education	16 (29.6%)	27 (56.3%)
Higher education	32 (59.3%)	14 (29.2%)
Missing	3 (5.3%)	7 (12.7%)
CIPN Onset > 3 months	57 (100%)	55 (100%)
MPI pain interference	2.4 (1.5)	2.7 (1.6)
NRS pain intensity (average days)	5.5 (1.5)	5.5 (1.7)
NRS pain intensity (worst days)	7.2 (1.5)	7.2 (1.7)
CIPN Total mean score	1.3 (0.5)	1.3 (0.6)
CIPN Subscale sensory symptoms	1.3 (0.5)	1.3 (0.7)
CIPN Subscale motor symptoms	1.2 (0.6)	1.2 (0.6)
CIPN Subscale autonomic symptoms	1.4 (0.9)	1.3 (0.9)
ELS value‐based living total mean score	3.6 (0.7)	3.3 (0.7)
**ELS subscale valued living**	**3.9 (0.6)**	**3.6 (0.7)**
ELS subscale life fulfilment	3.2 (1)	2.9 (0.9)
HADS psychological distress mean score	0.8 (0.5)	0.7 (0.5)
HADS subscale anxiety symptoms	0.8 (0.6)	0.8 (0.5)
HADS subscale depressive symptoms	0.7 (0.5)	0.7 (0.5)
PCS pain catastrophizing total mean score	1.3 (0.8)	1 (0.8)
PCS subscale rumination	1.6 (1.0)	1.3 (1.0)
PCS subscale magnification	0.9 (0.8)	0.6 (0.7)
**PCS subscale helplessness**	**1.3 (0.8)**	**0.9 (0.8)**
PIPS psychological flexibility total mean score	2.7 (1)	2.7 (1)
PIPS subscale avoidance	2.1 (1.2)	2.1 (1.1)
PIPS subscale cognitive fusion	3.9 (1)	3.8 (1.2)
QLQ‐C30 global health status	63.3 (16.4)	65.8 (17.6)
QLQ‐C30 physical functioning	66.7 (17.4)	67.2 (19)
QLQ‐C30 role functioning	62 (25.8)	57.6 (26.2)
QLQ‐C30 emotional functioning	74.4 (25.6)	77.6 (19.1)
QLQ‐C30 cognitive functioning	70.1 (26.2)	64.6 (26.8)
QLQ‐C30 social functioning	72.2 (24.4)	73.6 (24.8)
QLQ‐C30 fatigue	40.5 (25.9)	40.5 (22.3)
QLQ‐C30 nausea/vomiting	5.2 (16.5)	3.1 (8.9)
QLQ‐C30 pain	47.8 (20.5)	46.5 (24.8)
QLQ‐C30 dyspnoea	17.3 (21.2)	15.3 (23.8)
QLQ‐C30 insomnia	41.4 (33)	38.2 (37.7)
QLQ‐C30 appetite loss	8 (19.4)	8.3 (23.3)
QLQ‐C30 constipation	9.9 (19)	19.4 (29.8)
QLQ‐C30 diarrhea	5.6 (14.1)	9.7 (21.7)
QLQ‐C30 financial problems	13.6 (23.8)	12.5 (25.4)

*Note:* Bold‐faced characteristics show statistically significantly differences between the treatment groups. Mean (SD) reported for continuous variables and *N* (%) for categorical variables.

### 3.1 Effectiveness of the Online ACT Intervention

A series of linear mixed models was fitted to investigate whether the ACT and WLC groups differed in change over time on the primary and secondary outcomes. These models tested group differences in change from baseline (T0) to the 3‐month follow‐up (T4), as reflected in the Treatment ∗ Time interaction. Table 2 shows the fixed effects *F*‐tests for Time and the Time ∗ Treatment interaction across all outcomes. For the prespecified primary outcome, pain interference (MPI–Interference), no significant Treatment ∗ Time interaction was observed, F (2, 77.95) = 0.28, *p* = 0.758, indicating that changes in pain interference over time did not differ significantly between the ACT and WLC groups. Thus, the primary study hypothesis was not supported.

**TABLE 2:** Effects of the online ACT intervention versus waiting list control on primary and secondary outcomes: fixed effects estimates from linear mixed models.

Outcome	Time	*p*‐value	Time ∗ Treatment	*p*‐value
MPI pain interference	*F* (2, 77.95) = 6.53	**0.002** *	*F* (2, 77.95) = 0.28	0.758
QLQ‐C30 global health status	*F* (2, 77.39) = 2.2	0.117	*F* (2, 77.39) = 2.44	0.094
QLQ‐C30 physical functioning	*F* (2, 78.28) = 4.19	0.019*	*F* (2, 78.28) = 0.42	0.662
QLQ‐C30 role functioning	*F* (2, 73.80) = 0.34	0.713	*F* (2, 73.80) = 1.84	0.166
QLQ‐C30 emotional functioning	*F* (2, 77.61) = 3.62	0.032*	*F* (2, 77.61) = 0.7	0.501
QLQ‐C30 cognitive functioning	*F* (2, 81.41) = 7.84	**< 0.001** *	*F* (2, 81.41) = 0.19	0.831
QLQ‐C30 social functioning	*F* (2, 80.16) = 1.35	0.266	*F* (2, 80.16) = 2.01	0.141
CIPN symptom severity mean	*F* (2, 76.72) = 8.17	**< 0.001** *	*F* (2, 76.72) = 0.6	0.554
CIPN subscale sensory symptoms	*F* (2, 74.61) = 0.29	0.752	*F* (2, 74.61) = 0.14	0.875
CIPN subscale motor symptoms	*F* (2, 78.56) = 4.34	0.016*	*F* (2, 78.56) = 1.5	0.230
CIPN subscale autonomic symptoms	*F* (2, 80.03) = 10.67	**< 0.001** *	*F* (2, 80.03) = 1.2	0.307
NRS pain intensity (average)	*F* (2, 76.56) = 12.26	**< 0.001** *	*F* (2, 76.56) = 0.28	0.758
NRS pain intensity (worst)	*F* (2, 76.58) = 13.92	**< 0.001** *	*F* (2, 76.58) = 0.91	0.405
HADS psychological distress mean	*F* (2, 80.20) = 1.11	0.334	*F* (2, 80.20) = 0.8	0.453
HADS subscale anxiety	*F* (2, 78.89) = 1.48	0.233	*F* (2, 78.89) = 1.89	0.158
HADS subscale depression	*F* (2, 79.95) = 0.49	0.615	*F* (2, 79.95) = 0.11	0.892
PCS pain catastrophizing mean	*F* (2, 76.55) = 18.05	**< 0.001** *	*F* (2, 76.55) = 7.2	**0.001** *
PCS subscale rumination	*F* (2, 78.53) = 13.53	**< 0.001** *	*F* (2, 78.53) = 2.32	0.105
PCS subscale magnification	*F* (2, 81.36) = 6.91	**0.002** *	*F* (2, 81.36) = 3.23	0.045*
PCS subscale helplessness	*F* (2, 78.57) = 15.26	**< 0.001** *	*F* (2, 78.57) = 8.53	**< 0.001** *
PIPS psychological flexibility mean	*F* (2, 80.91) = 14.89	**< 0.001** *	*F* (2, 80.91) = 1.09	0.341
PIPS subscale avoidance	*F* (2, 79.62) = 9.37	**< 0.001** *	*F* (2, 79.62) = 0.83	0.438
PIPS subscale cognitive fusion	*F* (2, 84.77) = 18.77	**< 0.001** *	*F* (2, 84.77) = 0.63	0.536
FMI mindfulness inventory	*F* (2, 82.94) = 2.18	0.119	*F* (2, 82.94) = 1.35	0.264
ELS value‐based living mean	*F* (2, 81.68) = 2.55	0.085	*F* (2, 81.68) = 0.19	0.826
ELS subscale valued living	*F* (2, 81.84) = 1.33	0.271	*F* (2, 81.84) = 0.67	0.513
ELS subscale life fulfilment	*F* (2, 81.24) = 2.63	0.078	*F* (2, 81.24) = 0.34	0.714

*Note:* Bold‐faced effects remain statistically significantly after a Holm‐Bonferroni correction.

^*^ *p* < 0.05.

With respect to secondary outcomes, a significant Treatment * Time interaction was observed for the PCS total score *F* (2, 76.55) = 7.20, *p* = 0.001, indicating greater reductions in pain catastrophizing in the ACT group compared with the WLC group (Table 2). Detailed results for the PCS subscales are presented in Supporting Information S1: Table S3, while Table 3 presents the corresponding standardized effect sizes (Cohen's d) at post‐treatment and 3‐month follow‐up. These findings indicate that participants in the ACT group experienced greater reductions in pain catastrophizing than participants in the WLC group at both post‐treatment and the 3‐month follow‐up. As pain catastrophizing was a secondary outcome, this observation should be considered exploratory rather than evidence that the primary efficacy objective was met.

**TABLE 3:** Estimated Cohen's *d* effect sizes (95% CI) expressing the model‐based standardized mean difference between the ACT and WLC groups on the primary and secondary outcomes, both at post‐treatment (T3) and 3‐month follow‐up (T4).

Outcome	Cohen's *d* (T3)	Cohen's *d* (T4)
MPI pain interference	0.01 (−0.43, 0.45)	−0.17 (−0.62, 0.28)
QLQ‐C30 global health status	0.36 (−0.08, 0.8)	−0.12 (−0.57, 0.33)
QLQ‐C30 physical functioning	0.13 (−0.31, 0.57)	0.21 (−0.24, 0.66)
QLQ‐C30 role functioning	0.02 (−0.42, 0.46)	−0.1 (−0.55, 0.35)
QLQ‐C30 emotional functioning	0.33 (−0.11, 0.77)	0.44 (−0.02, 0.9)
QLQ‐C30 cognitive functioning	−0.13 (−0.57, 0.31)	−0.03 (−0.48, 0.42)
QLQ‐C30 social functioning	0.35 (−0.09, 0.79)	0.46 (0, 0.92)
CIPN symptom severity mean	−0.08 (−0.52, 0.36)	−0.3 (−0.75, 0.15)
CIPN subscale sensory symptoms	−0.06 (−0.5, 0.38)	−0.07 (−0.52, 0.38)
CIPN subscale motor symptoms	−0.39 (−0.83, 0.05)	−0.28 (−0.73, 0.17)
CIPN subscale autonomic symptoms	0.16 (−0.28, 0.6)	−0.26 (−0.71, 0.19)
NRS pain intensity (average days)	0.16 (−0.28, 0.6)	0.16 (−0.29, 0.61)
NRS pain intensity (worst days)	0.28 (−0.16, 0.72)	0.19 (−0.26, 0.64)
HADS psychological distress mean	−0.07 (−0.51, 0.37)	−0.31 (−0.76, 0.14)
HADS subscale anxiety	0.02 (−0.42, 0.46)	−0.16 (−0.61, 0.29)
HADS subscale depression	−0.12 (−0.56, 0.32)	−0.43 (−0.88, 0.02)
PCS pain catastrophizing mean	**−0.71 (−1.16, −0.26)** *	**−0.57 (−1.03, −0.11)** *
PCS subscale rumination	−0.34 (−0.78, 0.1)	−0.32 (−0.77, 0.13)
PCS subscale magnification	−0.45 (−0.89, −0.01)*	−0.29 (−0.74, 0.16)
PCS subscale helplessness	**−0.74 (−1.19, −0.29)** *	**−0.65 (−1.11, −0.19)** *
PIPS psychological flexibility mean	−0.3 (−0.74, 0.14)	−0.06 (−0.51, 0.39)
PIPS subscale avoidance	−0.29 (−0.73, 0.15)	−0.11 (−0.56, 0.34)
PIPS subscale cognitive fusion	−0.19 (−0.63, 0.25)	0.04 (−0.41, 0.49)
FMI mindfulness inventory	0.01 (−0.43, 0.45)	0.45 (−0.01, 0.91)
ELS value‐based living mean	0.17 (−0.27, 0.61)	0.03 (−0.42, 0.48)
ELS subscale valued living	0.07 (−0.37, 0.51)	−0.06 (−0.51, 0.39)
ELS subscale life fulfilment	0.3 (−0.14, 0.74)	0.19 (−0.26, 0.64)

*Note:* Positive estimates indicate higher mean scores in the ACT group than in the WLC group. Bold‐faced effects remain statistically significantly after a Holm‐Bonferroni correction.

^*^ *p* < 0.05.

Table 2 also reports the main effects of Time for all primary and secondary outcomes, reflecting overall changes across the follow‐up period regardless of treatment condition. Significant improvements over time were observed for the primary outcome, MPI pain interference, F (2, 77.95) = 6.53, *p* = 0.002. Significant time effects were also found for CIPN symptom severity, F (2, 76.72) = 8.17, *p* < 0.001; NRS pain intensity (average days), F (2, 76.56) = 12.26, *p* < 0.001; NRS pain intensity (worst days), F (2, 76.80) = 13.92, *p* < 0.001; PCS pain catastrophizing, F (2, 76.55) = 18.05, *p* < 0.001; and PIPS psychological flexibility, F (2, 80.91) = 14.89, *p* < 0.001. Detailed results for individual subscales and pairwise comparisons across timepoints are reported in Supporting Information S1: Table S3. These analyses indicate that improvements on most outcomes were maintained from post‐treatment to the 3‐month follow‐up.

### 3.2 Clinical Improvement

Table 4 shows the proportion of participants in the ACT and WLC group who showed clinically significant improvement, at either post‐treatment or 3‐month follow‐up, on MPI pain interference, NRS pain intensity (average or worst pain), and CIPN symptom severity. For the primary outcome, no between‐group difference in clinically significant improvement was observed at post‐treatment. However, at 3‐month follow‐up, a significantly larger proportion of participants in the ACT group (47.4%) met criteria for clinical improvement compared with the WLC group (23.6%). In contrast, deterioration of > 1 point on MPI pain interference occurred in 13.5% of ACT participants versus 5.1% of WLC participants. Because pain interference reflects the extent to which pain disrupts daily functioning and activities, this deterioration should be considered clinically meaningful rather than merely reflecting fluctuations in symptom severity. Similar results were found in the completers‐only analysis. After applying Holm‐Bonferroni correction for multiple testing, no significant between‐group differences were found for clinical improvement on NRS pain intensity and CIPN symptom severity, in either the imputed or the completers‐only analyses. Patients in the ACT group were also asked to report on their average weekly engagement with the online self‐help training. At post‐test, 19 of 57 patients provided no response, and 7 of 57 reported engaging less than the required ≥ 2 hours per week. A sensitivity analysis excluding these 26 participants did not alter the conclusions of our primary and secondary analyses (Tables 2 and 4).

**TABLE 4:** The number (%) of participants in each group with clinically significant improvement in the outcomes pain interference (MPI), pain intensity (NRS) and symptom severity (CIPN) at post‐treatment (T3) and 3‐month follow‐up (T4).

Outcome	Online self‐help (ACT)	Waiting list control (WLC)	*p*‐value
Expectation maximization imputation			
Pain interference (MPI)			
Post‐treatment (T3)	17 (29.8%)	13 (23.6%)	0.460
3‐month follow‐up (T4)	27 (47.4%)	13 (23.6%)	**0.009** *
Pain intensity (NRS) average days			
Post‐ treatment (T3)	25 (43.9%)	26 (47.3%)	0.717
3‐month follow‐up (T4)	26 (45.6%)	32 (58.2%)	0.183
Pain intensity (NRS) worst days			
Post‐ treatment (T3)	20 (35.1%)	23 (41.8%)	0.464
3‐month follow‐up (T4)	20 (35.1%)	30 (54.5%)	0.038*
CIPN Symptom severity			
Post‐ treatment (T3)	22 (38.6%)	16 (29.1%)	0.288
3 month follow‐up (T4)	24 (42.1%)	19 (34.5%)	0.411
Completers only analysis			
Pain interference (MPI)			
Post‐ treatment (T3)	12 (38.7%)	8 (21.2%)	0.108
3‐month follow‐up (T4)	16 (51.6%)	8 (21.2%)	**0.008** *
Pain intensity (NRS) average days			
Post‐ treatment (T3)	12 (38.7%)	18 (47.4%)	0.470
3‐month follow‐up (T4)	15 (48.4%)	23 (60.5%)	0.313
Pain intensity (NRS) worst days			
Post‐ treatment (T3)	9 (29.0%)	17 (44.7%)	0.181
3‐month follow‐up (T4)	10 (32.3%)	19 (50.0%)	0.138
CIPN Symptom severity			
Post‐ treatment (T3)	14 (45.2%)	10 (26.3%)	0.102
3‐month follow‐up (T4)	17 (54.8%)	13 (34.2%)	0.086

*Note:* Bold‐faced p‐values remain statistically significant after Holm‐Bonferroni correction. In line with protocol, missing scores of dropouts were imputed with expectation maximization. As a sensitivity check, the same statistics are reported for completers only.

^*^ *p* < 0.05. *p*‐values are obtained from a chi‐square test of the null hypothesis that the proportion of patients with clinical significance is independent of the treatment group.

### 3.3 Predictors of Treatment Effectiveness

The second objective was to identify baseline predictors of the treatment effectiveness on the primary outcome, MPI pain interference. Supporting Information S1: Table S4 presents the ANCOVA results testing whether baseline characteristics moderated the extent to which ACT improved MPI pain interference more than WLC from baseline to post‐treatment (T3) or 3‐month follow‐up (T4). The Table also shows the main effects of each baseline characteristic, reflecting predictors of overall improvement regardless of treatment condition. None of the preregistered baseline variables, including demographic, clinical, and psychological factors, significantly moderated the treatment effect.

### 3.4 Guided Versus Unguided Self‐Help Intervention

At 3‐month follow‐up, the 38 remaining patients in the WLC group were offered access to the unguided version of the online ACT self‐help intervention. The third objective was to compare their change from 3‐month follow‐up (T4) to post‐treatment (6‐month follow‐up (T5)) with the change observed in the guided ACT group from baseline (T0) to post‐treatment (T3). Table 5 shows the estimated Time ∗ Treatment effects, testing differences between guided and unguided ACT in pre‐post change on all outcomes. No statistically significant differences were observed between guided and unguided ACT regarding change on the primary outcome, MPI pain interference. However, after applying Holm‐Bonferroni correction for multiple testing, patients in the guided ACT group demonstrated significantly greater improvements in the secondary outcomes PCS pain catastrophizing and PIPS psychological flexibility compared to those in the unguided group. Similar improvements were observed across all PCS and PIPS subscales.

**TABLE 5:** Guided versus unguided ACT. Estimated unstandardized regression coefficients (SE) and *F*‐test of the Time ∗ Treatment effect, testing the difference between guided and unguided ACT in the change from pre‐to post‐treatment on various outcomes.

Outcome	B (SE)	F (df1, df2)	*p*‐value
MPI pain interference	−0.49 (0.37)	*F* (1, 73.47) = 1.77	0.187
QLQ‐C30 global health status	5.95 (4.18)	*F* (1, 71.58) = 2.02	0.159
QLQ‐C30 physical functioning	2.01 (2.58)	*F* (1, 69.50) = 0.61	0.439
QLQ‐C30 role functioning	11.64 (5.05)	*F* (1, 72.46) = 5.32	0.024*
QLQ‐C30 emotional functioning	−1.66 (4.77)	*F* (1, 73.48) = 0.12	0.729
QLQ‐C30 cognitive functioning	4.70 (3.97)	*F* (1, 72.36) = 1.41	0.240
QLQ‐C30 social functioning	4.46 (4.51)	*F* (1, 75.27) = 0.98	0.326
CIPN symptom severity mean	−0.10 (0.10)	*F* (1, 69.27) = 1.11	0.296
CIPN subscale sensory symptoms	0.06 (0.10)	*F* (1, 69.61) = 0.28	0.602
CIPN subscale motor symptoms	−0.21 (0.10)	*F* (1, 71.98) = 4.61	0.035*
CIPN subscale autonomic symptoms	−0.13 (0.17)	*F* (1, 70.61) = 0.56	0.457
NRS pain intensity (average days)	−0.91 (0.39)	*F* (1, 70.85) = 5.41	0.023*
NRS pain intensity (worst days)	−1.11 (0.56)	*F* (1, 77.82) = 3.97	0.050
HADS psychological distress mean	0.16 (0.08)	*F* (1, 71.60) = 3.71	0.058
HADS subscale anxiety	0.10 (0.07)	*F* (1, 68.78) = 2.36	0.129
HADS subscale depression	0.04 (0.08)	*F* (1, 68.03) = 0.20	0.657
PCS pain catastrophizing mean	−0.57 (0.11)	*F* (1, 72.14) = 27.97	**< 0.001** *
PCS subscale rumination	−0.71 (0.17)	*F* (1, 74.20) = 17.85	**< 0.001** *
PCS subscale magnification	−0.33 (0.10)	*F* (1, 70.26) = 11.68	**0.001** *
PCS subscale helplessness	−0.60 (0.13)	*F* (1, 73.72) = 21.09	**< 0.001** *
PIPS psychological flexibility mean	−0.82 (0.20)	*F* (1, 74.08) = 17.33	**< 0.001** *
PIPS subscale avoidance	−0.72 (0.22)	*F* (1, 73.24) = 10.78	**0.002** *
PIPS subscale cognitive fusion	−0.99 (0.29)	*F* (1, 76.95) = 11.90	**< 0.001** *
FMI mindfulness inventory	−0.05 (0.08)	*F* (1, 75.16) = 0.29	0.594
ELS value‐based living mean	0.15 (0.10)	*F* (1, 75.24) = 2.18	0.144
ELS subscale valued living	0.11 (0.10)	*F* (1, 78.98) = 1.17	0.283
ELS subscale life fulfilment	0.22 (0.14)	*F* (1, 72.99) = 2.40	0.126

*Note:* Negative estimates indicate larger reductions in the ACT group than in the WLC group. Bold‐faced *p*‐values remained statistically significant after a Holm‐Bonferroni correction.

^*^ *p* < 0.05.

## 4 Discussion

Chronic painful CIPN is a highly debilitating long‐term consequence of chemotherapy that affects many adult cancer survivors and substantially reduces their QoL. Since treatment options are limited, online self‐help interventions may offer a valuable form of support. This RCT evaluated the effectiveness of an online ACT self‐help intervention for reducing pain interference in cancer survivors with chronic painful CIPN, compared to a WLC. The primary outcome analyses showed no significant treatment effect on pain interference, as ACT did not outperform the WLC over time. This finding suggests that the intervention, in its current form, cannot be considered effective in reducing pain interference at the group level. An additional consideration is that eligibility was based on the presence of painful CIPN and a minimum pain intensity level, rather than a minimum level of pain interference. Consequently, some participants may have experienced substantial pain but relatively limited interference with daily functioning at baseline, thereby limiting the potential for improvement on the primary outcome. Future studies may benefit from including a minimum pain interference threshold when pain interference is the primary endpoint.

Although no significant treatment effect was observed for the primary outcome, exploratory findings provide some indications of potential benefit. A larger proportion of ACT participants showed clinically significant improvement in pain interference at 3‐month follow‐up (47.4%) compared with WLC participants (23.6%). Importantly, pain interference reflects the impact of pain on daily functioning rather than pain severity alone. Therefore, deterioration in this outcome could also be regarded as clinically meaningful. At the same time, slightly more ACT participants showed deterioration in pain interference (> 1‐point increase; 13.5% vs. 5.1% in WLC), suggesting substantial heterogeneity in response. Several mechanisms may explain this deterioration. Some participants may have developed increased self‐awareness of pain without sufficient progress in acceptance or behavior change, potentially heightening hypervigilance and avoidance patterns [53, 54]. Others may have struggled to maintain behavioral changes after completing the guided phase of the intervention. Additionally, unmet expectations regarding the intervention's effects may have contributed to a nocebo‐like response, amplifying perceived pain or functional limitations [55]. The above‐mentioned explanations remain speculative, but the findings highlight the importance of systematically monitoring both improvement and deterioration in future ACT studies for painful CIPN.

Exploratory analyses further showed that ACT participants experienced greater reductions in pain catastrophizing than WLC participants, with medium to large effect sizes at both post‐treatment and 3‐month follow‐up. Pain catastrophizing is an important psychological correlate of chronic pain and has been associated with poorer adjustment and greater disability in previous studies [56, 57, 58]. Although reductions in pain catastrophizing have been reported in ACT studies, catastrophizing is not considered a direct measure of ACT‐related mechanism change. Therefore, the observed reductions in pain catastrophizing should not be interpreted as evidence that ACT exerted its effects through its proposed mechanisms. This interpretation is further supported by the fact that psychological flexibility did not improve significantly more in the ACT group and that mediation analyses were not conducted. Future research could investigate whether changes in catastrophizing and related psychological processes explain improvements in pain interference.

Although no group differences were observed for other secondary outcomes, patients in both conditions showed overall improvement in pain interference, CIPN symptom severity, pain intensity, and psychological flexibility. Several factors may explain these general improvements. Participation in the study may have provided recognition and validation of symptoms, something CIPN patients often report lacking, as they are frequently told to simply “learn to live with” their condition without acknowledgment of the condition's complexity [59, 60]. Furthermore, participation in a study emphasizing symptom acceptance may enhance self‐awareness and promote reflection, thereby altering the way individuals relate to their symptoms.

No baseline variables—including age, sex, education, comorbidities, time since CIPN onset, symptom severity, quality of life, pain intensity, depression, anxiety, psychological inflexibility, mindfulness, value‐based living, or pain catastrophizing—moderated treatment effects on pain interference. This aligns with prior research showing that few robust moderators have been identified in psychosocial interventions for chronic pain [61, 62]. While many participants showed clinically meaningful improvements, a considerable subset did not, and the overall ACT versus waitlist effect on pain interference was not significant. This indicates heterogeneous responses despite the absence of identifiable baseline moderators, suggesting some participants might have benefited significantly from the intervention, while others not so much. Larger future studies are needed to more definitively determine which patients benefit most from ACT‐based self‐help interventions.

The third objective was to examine whether effects differed between guided and unguided versions of the online ACT self‐help intervention. Patients who received guided ACT demonstrated greater improvements from baseline to post‐treatment in pain catastrophizing and psychological flexibility than participants from the WLC group who later crossed over to receive the unguided version of the intervention (*n* = 38). This may indicate that guidance facilitates changes in psychological flexibility [18, 63] and may also contribute to improvements in broader pain‐related cognitive processes such as pain catastrophizing [53, 64]. The findings align with previous systematic reviews and meta‐analyses showing that guided digital interventions generally produce better outcomes, particularly regarding adherence and engagement [32, 65, 66]. Evidence on cost‐effectiveness remains mixed; for example, one RCT found no economic advantage of guided over unguided versions [67]. As a recent review noted, results regarding the added value of guidance are not uniformly consistent [65]. In the present study, the guidance offered was intentionally low‐threshold and optional, reflecting both practical constraints and exploratory interviews that revealed diverse participant preferences [17]. More structured formats, such as asynchronous email guidance [23] or personalized standardized feedback [68] could have provided greater continuity of support, but flexibility was prioritized in the study. Future implementations should carefully tailor the type and intensity of guidance with the needs of specific patient groups and the therapeutic goals of the intervention.

### 4.1 Implications for Clinical Practice

The current study provides an entry point for the development of psychological interventions for cancer patients experiencing pain. Online self‐help interventions may be particularly promising in this context; however, they are unlikely to be suitable for all patients. For individuals with high symptom burden, a purely self‐guided format may not be feasible or sufficient, underscoring the importance of flexible intervention designs that allow patients to choose whether, how, and to what extent they receive professional support. Such interventions should be positioned as an addition to conventional care pathways. They may be especially useful during waiting list periods or as a low‐intensity resource for individuals with relatively mild symptoms. Ultimately, healthcare professionals must determine which type of care is most appropriate for which patients, and at what point in the trajectory. This also applies to online psychosocial interventions such as the one examined in the current study. The Embrace Pain intervention is available for use in future studies and can be accessed by other researchers who wish to evaluate or further develop the program in different populations or settings.

### 4.2 Limitations and Strengths

Some limitations need to be mentioned. First, although the trial was conducted as planned, recruitment proved more challenging than anticipated. With 112 patients included, our sample was 23% smaller than the 146 patients required according to our power analysis. Consequently, statistical power was lower than originally intended, particularly for detecting smaller treatment effects. As a result, although this remains possible, the absence of a significant treatment effect on the primary outcome should not be interpreted as definitive evidence of no effect. The sample size calculation was based on an anticipated effect size of *d* = 0.50 derived from previous ACT studies in chronic pain populations. Although justified by the available literature at the time, this assumption may have been optimistic for an online intervention targeting painful CIPN. Smaller treatment effects may in fact be more realistic in this population, possibly because patients with painful CIPN often experience fatigue and cognitive difficulties related to cancer and its treatment, which may make it more difficult to engage with an intervention requiring concentration, reflection, and the processing of information. Even so, smaller improvements may still be clinically meaningful as painful CIPN is a chronic condition with few evidence‐based treatment options, and modest reductions in symptoms or their impact on daily functioning may represent worthwhile benefits for patients. Previous research showed that poor recruitment was often due to, among other things, narrow eligibility criteria and a high burden, for example through the requirement to complete multiple and relatively lengthy questionnaires, which could also apply to the current RCT [69]. Furthermore, during recruitment we observed that patients frequently thought they did not qualify for the study because they did not perceive their symptoms to be “painful,” while the description of the sensations they experienced fitted the semantic approach to the word pain. A possible explanation is that individuals may have difficulty recognizing nerve pain due to limited exposure to nerve pain in childhood, combined with the fact that the understanding of pain is largely shaped by early verbal communication about personal experiences [70, 71, 72]. Additionally, it was noticed throughout the project that many people downplay the seriousness of their symptoms in the context of the life‐threatening disease cancer they have or had. Thereby, some patients do not perceive CIPN as a major burden, even though they experience symptoms and resulting limitations. They tend to downplay the severity of their symptoms, which is also likely to make them less likely to seek care. However, this does not imply that these patients experience no limitations or could not potentially benefit from supportive interventions. As a result, it is plausible that there is a significant group of patients who did not participate even though they might have benefited from the training. Another limitation is that information regarding a formal clinician diagnosis of CIPN was not systematically collected. However, no universally accepted diagnostic gold standard for painful CIPN currently exists in survivorship research, and although eligibility was based on multiple criteria designed to maximize the likelihood that participants experienced painful CIPN, some degree of diagnostic uncertainty cannot be excluded. These challenges are not unique to the present study and reflect broader difficulties in defining and identifying neuropathic pain populations for research purposes.

Second, the method of inviting participants to the intervention changed during the study. During the study, we found out that particularly WLC participants did not activate their account when they received an invitation to do so. Therefore, to increase adherence, as of August 2023 all patients (both the active group and waiting list group) received a personal email announcing that they would receive an invitation to create an account in the online intervention.

Third, the reasons for dropout may not have been completely random. Both the intention‐to‐treat analyses using linear mixed models and the Expectation Maximization imputation used for the clinical improvement analyses assume that data are missing independently of the outcomes themselves. Compared to completers, dropouts were slightly younger and more often female, and generally reported higher fatigue, more depressive symptoms, and lower QoL at baseline. Although these differences did not remain statistically significant after Holm‐Bonferroni correction, and although Little's MCAR test was not significant, the power of these analyses may have been limited by the sample size and the number of variables examined. Therefore, the possibility that attrition affected the precision of the estimates and confidence in the findings cannot be completely excluded.

Fourth, not all patients randomized to the guided self‐help intervention made use of the available guidance. Given the low‐threshold and non‐mandatory nature of the guidance offered, participants were free to refrain from engaging with the guidance, which may have resulted in varying levels of uptake and exposure to therapist guidance. However, in accordance with intention‐to‐treat analysis, all participants in the ACT group were included in the analyses as “guided participants”, regardless of whether, or to what extent, they made use of the guidance. While potential non‐adherence may dilute intervention effects, the sensitivity analyses suggest that excluding non‐adherent participants did not alter the conclusions of our primary and secondary analyses. It is also possible that the intervention intensity was insufficient for some participants. Embrace Pain consisted of six online modules delivered over 8 weeks with optional asynchronous guidance and no scheduled therapist contact. Future studies should investigate whether a more intensive intervention format or more proactive guidance may improve outcomes for some patients.

This study also has several strengths. First, pain interference was included as the main outcome measure, which was recommended in a previous meta‐analysis on ACT for chronic pain [24]. Pain interference is considered an appropriate outcome measure for studies of ACT, given its alignment with the goal of this type of intervention which focuses on learning to live with pain and performing valuable activities despite pain. Second, this study focused on painful CIPN, which has been shown to result in worse QoL compared to non‐painful CIPN [16], making painful CIPN a more clinically relevant endpoint. Third, the intervention examined in this study was developed using a patient‐centered approach, which allowed for the final intervention to match as closely as possible to the specific needs and preferences of the user group [17]. This patient‐oriented design not only enhances the relevance and acceptability of the intervention but also increases the likelihood of successful implementation [73].

## 5 Conclusion

Chronic painful CIPN substantially impairs QoL and treatment options remain limited. This RCT showed that the online ACT‐based intervention did not significantly reduce pain interference compared with WLC. A notable proportion of participants in the ACT group nevertheless experienced clinically meaningful improvement, suggesting that some participants may have benefited from the intervention. The intervention was also associated with sustained reductions in pain catastrophizing, but this secondary finding should be considered exploratory and should not be interpreted as evidence of ACT‐specific mechanism change. Also, some participants showed a temporary worsening of pain interference. The reasons for this deterioration could not be determined from the available data and should be investigated in future research. Overall, these findings suggest that online ACT‐based self‐help offers may be promising for supporting this patient population and provide a basis for further research to optimize such interventions and clarify for whom and under which conditions they are most effective.

## Funding

This study was supported by the Dutch Cancer Society (#12181). The Dutch Cancer Society had no role in the study design, writing of the manuscript, or submission. Tilburg University is the sponsor of this study.

## Supporting information


Supporting Information S1

## Data Availability

The data that support the findings of this study are available on request from the corresponding author. The data are not publicly available due to privacy or ethical restrictions.
