# Supplementary material for: Embrace Pain—A Randomized Controlled Trial of Online Acceptance and Commitment Therapy for Painful Chemotherapy‐Induced Peripheral Neuropathy in Cancer Survivors
Source: Psychooncology. 2026 Sep 19;35(9):e70608. doi: 10.1002/pon.70608 (PMC13589260; doi:10.1002/pon.70608)
Supplement: Supplementary file 1 [file PON-35-e70608-s001.docx]

**Supplemental Table S1: Reliability estimates**

Estimated Cronbach’s alpha coefficients for all measurement scales at each time point.

| Measurement scale | T0a | T0b | T1 | T2 | T3 | T4 | T5 |
| --- | --- | --- | --- | --- | --- | --- | --- |
| MPI pain interference | x | 0.89 | 0.92 | 0.91 | 0.91 | 0.92 | 0.90 |
| CIPN mean score | 0.81 | x | x | x | 0.90 | 0.91 | 0.85 |
| QLQ-C30 global health status | x | 0.79 | x | x | 0.89 | 0.84 | 0.71 |
| QLQ-C30 physical functioning | x | 0.76 | x | x | 0.75 | 0.82 | 0.79 |
| QLQ-C30 role functioning | x | 0.83 | x | x | 0.89 | 0.90 | 0.86 |
| QLQ-C30 emotional functioning | x | 0.89 | x | x | 0.89 | 0.91 | 0.82 |
| QLQ-C30 cognitive functioning | x | 0.83 | x | x | 0.69 | 0.60 | 0.42 |
| QLQ-C30 social functioning | x | 0.87 | x | x | 0.86 | 0.88 | 0.74 |
| CIPN subscale sensory symptoms | 0.76 | x | x | x | 0.94 | 0.85 | 0.83 |
| CIPN subscale motor symptoms | 0.74 | x | x | x | 0.82 | 0.82 | 0.79 |
| CIPN subscale autonomic symptoms | **0.19** | x | x | x | **0.28** | **0.51** | **0.48** |
| ELS value-based living mean score | x | 0.94 | 0.93 | 0.95 | 0.96 | 0.96 | 0.95 |
| ELS subscale valued living | x | 0.91 | 0.91 | 0.93 | 0.95 | 0.95 | 0.92 |
| ELS subscale life fulfilment | x | 0.93 | 0.91 | 0.94 | 0.94 | 0.94 | 0.92 |
| FMI mindfulness mean score | x | 0.89 | 0.90 | 0.88 | 0.89 | 0.89 | 0.88 |
| HADS psychological distress | x | 0.90 | 0.86 | 0.89 | 0.91 | 0.90 | 0.90 |
| HADS anxiety subscale | x | 0.84 | 0.81 | 0.82 | 0.88 | 0.85 | 0.84 |
| HADS depression subscale | x | 0.81 | 0.79 | 0.85 | 0.84 | 0.83 | 0.86 |
| PCS subscale helplessness | x | 0.87 | 0.87 | 0.88 | 0.89 | 0.91 | 0.92 |
| PCS subscale rumination | x | 0.88 | 0.87 | 0.83 | 0.91 | 0.88 | 0.89 |
| PCS subscale magnification | x | 0.73 | 0.69 | 0.78 | 0.80 | 0.73 | 0.81 |
| PCS pain catastrophizing mean score | x | 0.93 | 0.92 | 0.93 | 0.95 | 0.94 | 0.95 |
| PIPS psychological flexibility mean score | 0.86 | x | 0.88 | 0.89 | 0.92 | 0.92 | 0.91 |
| PIPS subscale avoidance | 0.90 | x | 0.91 | 0.93 | 0.94 | 0.94 | 0.94 |
| PIPS subscale cognitive fusion | 0.72 | x | 0.75 | 0.77 | 0.74 | 0.81 | 0.80 |

*Note: bold-faced estimates indicate insufficient reliability based the criterion Cronbach’s alpha > 0.7*
*T0a: measured at inclusion*
*T0b: measured at baseline*

**Supplemental Table S2: Patient characteristics of dropouts and completers**

Differences in characteristics between participants who remained responding to the questionnaire until the last follow-up occasion (n=56) versus those who dropped out during follow-up (n=56).

| Patient characteristic | Completers (n=56) | Dropouts (n=56) | *p*-value |
| --- | --- | --- | --- |
| ACT intervention | 26 (46%) | 31 (55%) | .345 |
| Female Sex | 32 (57.1%) | 46 (82.1%) | .004* |
| Age | 65.8 (10.2) | 63.7 (11.6) | .036* |
| MPI pain interference | 2.4 (1.6) | 2.7 (1.5) | .241 |
| CIPN subscale sensory symptoms | 1.3 (0.6) | 1.4 (0.6) | .651 |
| CIPN subscale motor symptoms | 1.1 (0.5) | 1.2 (0.6) | .537 |
| CIPN subscale autonomic symptoms | 1.3 (0.8) | 1.4 (0.9) | .942 |
| ELS value-based living mean score | 3.6 (0.7) | 3.3 (0.7) | .073 |
| ELS subscale valued living | 3.8 (0.7) | 3.7 (0.6) | .239 |
| ELS subscale life fulfilment | 3.2 (1) | 2.8 (0.9) | .027* |
| HADS psychological distress mean score | 0.7 (0.4) | 0.9 (0.5) | .038 |
| HADS subscale anxiety symptoms | 0.7 (0.5) | 0.9 (0.6) | .081 |
| HADS subscale depressive symptoms | 0.6 (0.5) | 0.8 (0.5) | .033* |
| QLQ-C30 global health status | 68.8 (15) | 59.2 (17.8) | .004* |
| QLQ-C30 physical functioning | 67.3 (16.4) | 66.5 (20.1) | .838 |
| QLQ-C30 role functioning | 65.8 (23.9) | 52.9 (26.8) | .012* |
| QLQ-C30 emotional functioning | 79.5 (19.7) | 71.6 (25.5) | .08 |
| QLQ-C30 cognitive functioning | 72.6 (25.9) | 61.2 (26.1) | .03* |
| QLQ-C30 social functioning | 79.2 (21.1) | 65.2 (26.3) | .004* |
| QLQ-C30 fatigue | 35.3 (21.4) | 46.9 (26) | .016* |
| QLQ-C30 nausea / vomiting | 3.9 (13.1) | 4.7 (13.9) | .754 |
| QLQ-C30 pain | 45.5 (23.2) | 49.3 (21.6) | .406 |
| QLQ-C30 dyspnoea | 15.5 (22.9) | 17.4 (21.9) | .669 |
| QLQ-C30 insomnia | 35.7 (37.5) | 44.9 (31.6) | .189 |
| QLQ-C30 appetite loss | 8.3 (24.8) | 8 (16) | .932 |
| QLQ-C30 constipation | 16.1 (26.2) | 12.3 (23.7) | .454 |
| QLQ-C30 diarrhoea | 7.7 (18) | 7.2 (18.5) | .892 |
| QLQ-C30 financial problems | 9.5 (22.7) | 17.4 (26) | .106 |

*Mean (SD) reported for continuous variables and n (%) for categorical variables.
* p-value < .05. No p-values remained statistically significant after Holm–Bonferroni correction.*

**Supplemental Table 3: Estimated regression coefficients**

Estimated unstandardized regression coefficients (SE) for fixed effects of Treatment, Time and Time*Treatment.

| Outcome | Intercept (ACT T0) | Treatment (WLC) | Time  (T3) | Time  (T4) | Treatment (WLC) * Time (T3) | Treatment (WLC) * Time (T4) |
| --- | --- | --- | --- | --- | --- | --- |
| MPI pain interference | **2.41 (0.22)*** | 0.24 (0.32) | **-0.46 (0.22)*** | **-0.49 (0.24)*** | -.16 (0.31) | 0.06 (0.34) |
| QLQ-C30 global health status | **63.27 (2.31)*** | 2.53 (3.36) | 0.43 (2.48) | 0.30 (2.38) | -4.96 (3.48) | 1.91 (3.35) |
| QLQ-C30 physical functioning | **66.67 (2.47)*** | 0.56 (3.61) | **3.73 (1.55)*** | **3.98 (1.85)*** | -1.57 (2.18) | -2.18 (2.59) |
| QLQ-C30 role functioning | **74.38 (3.10)*** | 3.22 (4.52) | 1.57 (2.36) | 3.01 (2.91) | -5.72 (3.33) | -6.78 (4.10) |
| QLQ-C30 emotional functioning | **70.06 (3.60)*** | -5.48 (5.25) | 2.61 (2.42) | 1.18 (2.60) | 4.02 (3.43) | 2.83 (3.66) |
| QLQ-C30 cognitive functioning | **62.04 (3.53)*** | -4.40 (5.15) | **6.86 (2.92)*** | 6.00 (3.16) | 0.85 (4.10) | 2.67 (4.44) |
| QLQ-C30 social functioning | **72.22 (3.35)*** | 1.39 (4.88) | **6.47 (2.82)*** | 5.67 (3.04) | -6.41 (3.98) | -7.81 (4.28) |
| CIPN symptom severity mean | **1.28 (0.07)*** | -0.02 (0.11) | -0.11 (0.07) | **-0.24 (0.07)*** | 0.03 (0.10) | 0.10 (0.10) |
| CIPN subscale sensory symptoms | **1.31 (0.08)*** | 0.00 (0.11) | -0.01 (0.07) | -0.03 (0.08) | 0.04 (0.10) | 0.00 (0.12) |
| CIPN subscale motor symptoms | **1.19 (0.08)*** | -0.04 (0.11) | **-0.17 (0.07)*** | **-0.19 (0.07)*** | 0.15 (0.09) | 0.11 (0.09) |
| CIPN subscale autonomic symptoms | **1.35 (0.11)*** | -0.02 (0.16) | -0.12 (0.13) | **-0.49 (0.13)*** | -0.13 (0.18) | 0.16 (0.18) |
| NRS pain intensity (average days) | **5.53 (0.21)*** | -0.04 (0.31) | **-0.70 (0.30)*** | **-0.95 (0.32)*** | -0.29 (0.42) | -0.31 (0.45) |
| NRS pain intensity (worst days) | **7.16 (0.21)*** | -0.01 (0.30) | **-0.68 (0.34)*** | **-1.23 (0.40)*** | -0.64 (0.48) | -0.54 (0.57) |
| HADS psychological distress mean | **0.76 (0.07)*** | -0.01 (0.10) | 0.03 (0.05) | -0.03 (0.06) | 0.03 (0.07) | 0.10 (0.08) |
| HADS subscale anxiety | **0.83 (0.07)*** | -0.06 (0.11) | 0.04 (0.06) | -0.02 (0.06) | 0.07 (0.09) | 0.17 (0.09) |
| HADS subscale depression | **0.68 (0.07)*** | 0.04 (0.10) | 0.02 (0.05) | -0.04 (0.07) | 0.00 (0.07) | 0.04 (0.09) |
| PCS pain catastrophizing mean | **1.29 (0.10)*** | -0.30 (0.15) | **-0.48 (0.08)*** | **-0.51 (0.09)*** | **0.40 (0.11)*** | **0.34 (0.12)*** |
| PCS subscale rumination | **1.58 (0.13)*** | -0.25 (0.19) | **-0.55 (0.12)*** | **-0.56 (0.13)*** | **0.35 (0.17)*** | 0.30 (0.18) |
| PCS subscale magnification | **0.87 (0.10)*** | -0.23 (0.15) | **-0.28 (0.07)*** | **-0.31 (0.09)*** | **0.24 (0.10)*** | 0.23 (0.12) |
| PCS subscale helplessness | **1.29 (0.10)*** | **-0.32 (0.15)*** | **-0.44 (0.07)*** | **-0.53 (0.07)*** | **0.39 (0.10)*** | **0.37 (0.11)*** |
| PIPS psychological flexibility mean | **2.74 (0.13)*** | -0.06 (0.18) | **-0.62 (0.15)*** | **-0.65 (0.16)*** | 0.27 (0.21) | 0.10 (0.23) |
| PIPS subscale avoidance | **2.14 (0.16)*** | -0.05 (0.22) | **-0.52 (0.17)*** | **-0.59 (0.17)*** | 0.28 (0.24) | 0.14 (0.24) |
| PIPS subscale cognitive fusion | **3.93 (0.15)*** | -0.10 (0.21) | **-0.83 (0.17)*** | **-0.77 (0.23)*** | 0.24 (0.24) | 0.03 (0.32) |
| FMI mindfulness inventory | **2.82 (0.07)*** | -0.16 (0.11) | 0.06 (0.05) | 0.12 (0.07) | 0.03 (0.07) | -0.09 (0.10) |
| ELS value-based living mean | **3.62 (0.09)*** | **-0.32 (0.14)*** | 0.08 (0.06) | 0.07 (0.10) | 0.00 (0.08) | 0.08 (0.14) |
| ELS subscale valued living | **3.90 (0.09)*** | **-0.34 (0.13)*** | 0.04 (0.06) | 0.00 (0.10) | 0.06 (0.09) | 0.16 (0.14) |
| ELS subscale life fulfilment | **3.16 (0.13)*** | -0.30 (0.19) | 0.16 (0.08) | 0.19 (0.13) | -0.10 (0.12) | -0.05 (0.18) |

**** p < .05***

**Supplemental Table S4: predictors of treatment effect**

For various potential baseline predictors of the treatment effect on MPI pain interference, the unstandardized regression coefficients, F-statistics and p-values for both the predictor’s main effect and interactions with treatment.

| Predictor (X) | B (SE)  main effect X | F (df1, df2) | *p*-value | B (SE) X*Treatment | F (df1, df2) | *p*-value |
| --- | --- | --- | --- | --- | --- | --- |
| *Predictors of post-treatment (T3) MPI, controlling for pre-treatment (T0) MPI* | | | | |  |  |
| Female Sex | 0.298 (0.440) | F(1, 144)=1.12 | 0.294 | 0.064 (0.613) | F(1, 144)=0.01 | 0.919 |
| Age | 0.003 (0.018) | F(1, 144)=0.27 | 0.603 | -0.020 (0.026) | F(1, 144)=0.57 | 0.453 |
| Middle education (vs. High) | -0.362 (0.485) | F(2, 142)=0.02 | 0.976 | 0.874 (0.673) | F(2, 142)=1.19 | 0.311 |
| Lower education (vs. High) | -0.537 (0.599) | F(2, 142)=0.02 | 0.976 | 1.126 (0.889) | F(2, 142)=1.19 | 0.311 |
| CIPN symptom severity | 0.030 (0.023) | F(1, 144)=6.10 | 0.016* | 0.021 (0.029) | F(1, 144)=0.53 | 0.467 |
| QLQ-C30 Global health status | -0.005 (0.013) | F(1, 144)=1.66 | 0.201 | -0.018 (0.017) | F(1, 144)=1.01 | 0.318 |
| NRS pain intensity (average days) | -0.003 (0.141) | F(1, 144)=0.51 | 0.479 | 0.143 (0.180) | F(1, 144)=0.64 | 0.428 |
| NRS pain intensity (worst days) | -0.013 (0.132) | F(1, 144)=0.52 | 0.474 | 0.157 (0.175) | F(1, 144)=0.80 | 0.373 |
| HADS anxiety subscale | -0.090 (0.413) | F(1, 144)=1.36 | 0.247 | 0.932 (0.569) | F(1, 144)=2.68 | 0.106 |
| HADS depression subscale | 0.293 (0.503) | F(1, 144)=3.55 | 0.064 | 0.760 (0.610) | F(1, 144)=1.55 | 0.218 |
| PIPS psychological flexibility | 0.032 (0.231) | F(1, 144)=0.30 | 0.587 | 0.141 (0.312) | F(1, 144)=0.20 | 0.654 |
| FMI mindfulness inventory | 0.039 (0.392) | F(1, 144)=0.78 | 0.382 | -0.601 (0.563) | F(1, 144)=1.14 | 0.290 |
| ELS value-based living | -0.235 (0.308) | F(1, 144)=4.23 | 0.044* | -0.456 (0.400) | F(1, 144)=1.30 | 0.258 |
| PCS pain catastrophizing | -0.216 (0.278) | F(1, 144)=0.78 | 0.381 | 0.065 (0.374) | F(1, 144)=0.03 | 0.863 |
| *Predictors of MPI at 3-month follow-up (T4), controlling for pre-treatment (T0) MPI* | | | | |  |  |
| Female Sex | -0.754 (0.474) | F(1, 141)=0.20 | 0.659 | 1.209 (0.661) | F(1, 141)=3.35 | 0.071 |
| Age | 0.004 (0.023) | F(1, 141)=0.03 | 0.864 | -0.014 (0.031) | F(1, 141)=0.21 | 0.645 |
| Middle education (vs. High) | -0.656 (0.569) | F(2, 139)=0.17 | 0.844 | 0.977 (0.785) | F(2, 139)=0.78 | 0.461 |
| Lower education (vs. High) | -0.458 (0.650) | F(2, 139)=0.17 | 0.844 | 0.374 (0.990) | F(2, 139)=0.78 | 0.461 |
| CIPN symptom severity | -0.011 (0.028) | F(1, 141)=0.01 | 0.906 | 0.028 (0.034) | F(1, 141)=0.65 | 0.421 |
| QLQ-C30 Global health status | 0.012 (0.015) | F(1, 141)=0.10 | 0.754 | -0.016 (0.020) | F(1, 141)=0.70 | 0.407 |
| NRS pain intensity (average days) | 0.041 (0.162) | F(1, 141)=0.22 | 0.643 | 0.020 (0.199) | F(1, 141)=0.01 | 0.919 |
| NRS pain intensity (worst days) | 0.076 (0.155) | F(1, 141)=0.10 | 0.748 | -0.084 (0.194) | F(1, 141)=0.19 | 0.667 |
| HADS anxiety subscale | -0.567 (0.402) | F(1, 141)=0.24 | 0.629 | 0.807 (0.595) | F(1, 141)=1.84 | 0.180 |
| HADS depression subscale | 0.140 (0.531) | F(1, 141)=0.14 | 0.705 | 0.019 (0.677) | F(1, 141)=0.00 | 0.978 |
| PIPS psychological flexibility | 0.608 (0.264) | F(1, 141)=3.54 | 0.064 | -0.439 (0.343) | F(1, 141)=1.64 | 0.205 |
| FMI mindfulness inventory | 1.038 (0.428) | F(1, 141)=2.29 | 0.135 | -1.060 (0.609) | F(1, 141)=3.04 | 0.086 |
| ELS value-based living | 0.370 (0.398) | F(1, 141)=0.04 | 0.843 | -0.625 (0.478) | F(1, 141)=1.71 | 0.196 |
| PCS pain catastrophizing | -0.172 (0.297) | F(1, 141)=0.87 | 0.353 | -0.078 (0.406) | F(1, 141)=0.04 | 0.848 |

** p < .05. No p-values remained statistically significant after Holm–Bonferroni correction.*
